# Occurrence of Common Allergic Diseases in Children with Idiopathic Nephrotic Syndrome

**DOI:** 10.2188/jea.JE20140167

**Published:** 2015-05-05

**Authors:** Chang-Ching Wei, Cheng-Li Lin, Te-Chun Shen, Fung-Chang Sung

**Affiliations:** 1 Children’s Hospital, China Medical University Hospital, Taichung, Taiwan; 2 College of Medicine China Medical University, Taichung, Taiwan; 3 Management Office for Health Data, China Medical University Hospital, Taichung, Taiwan; 4 Department of Public Health, China Medical University, Taichung, Taiwan; 5 Division of Pulmonary and Critical Care Medicine, Department of Internal Medicine, Taichung, Taiwan; 6 Institute of Clinical Medical Science, China Medical University, Taichung, Taiwan

**Keywords:** allergic conjunctivitis, allergic rhinitis, asthma, atopic dermatitis, nephrotic syndrome

## Abstract

**Background:**

Clinical and immunological studies have consistently shown a possible link between atopy and idiopathic nephrotic syndrome (INS). However, whether allergic diseases occur after INS develops is unknown.

**Methods:**

From Taiwan’s National Health Insurance database, 1340 children with newly diagnosed INS and 5360 non-INS matched controls were identified in 2000–2007. By the end of 2008, the incidences and hazard ratios of four allergic diseases (allergic conjunctivitis, allergic rhinitis, atopic dermatitis, and asthma) were calculated.

**Results:**

The incidence rates of all four allergic diseases were greater in the INS cohort than in the non-INS cohort in all age groups and decreased sharply as age increased in both cohorts. Children with INS had the highest adjusted hazard ratio (4.13; 95% confidence interval [CI], 2.50–6.83) for atopic dermatitis and the lowest adjusted hazard ratio (1.71; 95% CI, 1.39–2.09) for allergic rhinitis. Most of the allergic diseases appeared within 2–6 months after INS developed, and the incidences declined with increasing follow-up duration.

**Conclusions:**

Allergic disorders are common in children with INS, especially within the first year after diagnosis. The role of INS in the development of allergic disorders should be elucidated to establish innovative disease intervention programs.

## INTRODUCTION

Idiopathic nephrotic syndrome (INS), the most common chronic kidney disease in children, has been reported to be associated with allergic disorders for more than 5 decades.^1^^–^^5^ Numerous studies have associated INS with the clinical and immunological features of allergies.^4^^–^^14^ From the 1950s through the 1970s, several anecdotal reports described patients who developed INS after allergic reactions to inhaled allergens, vaccinations, food, or insect stings.^1^^–^^3^ Meadow et al first reported a case-control study of 84 children with steroid-responsive nephrotic syndrome (NS) who had a greater incidence of atopic disorders, including asthma, eczema, recurrent urticaria, and hay fever than normal controls.^4^^,^^5^ In addition, their serum immunoglobulin E (IgE) levels tended to be increased, particularly in children with frequently relapsing nephrotic syndrome.^4^^,^^5^ Further, their first-degree relatives had an increased incidence of these atopic disorders.

Since 1970, increasing numbers of case-control studies have revealed elevated serum IgE levels and atopic diathesis in children with INS.^6^^–^^14^ Recently, levels of T-helper 2 (Th2)-associated cytokines, such as interleukins 4 and 13, were found to be elevated in patients with relapsing INS.^15^^–^^18^ Children with INS commonly have elevated serum levels of immunoglobulin E and Th2-associated cytokines. These immune responses also play a crucial role in allergic diseases.^19^^,^^20^ Although previous cross-sectional studies have extensively investigated the possible link between INS and atopic disorders, some potential problems, such as recall bias, limited patient numbers, and varying definitions of allergic disorders, should be considered in the interpretation of the inconsistent findings of the previous studies.

The first population-based cohort study of children with atopic dermatitis (AD) showed a greater incidence and risk of developing INS in this population.^22^ Atopic dermatitis, which is typically the first clinical manifestation of allergic disease, presents early in infancy, followed by the development of allergic rhinitis (AR) and asthma in some children; this is the so-called atopic march.^21^ Therefore, the atopic dermatitis cohort is an important model for investigating the impact of atopy on the development of INS. However, whether the increased risk of developing allergic diseases remains even after INS onset is unknown. If the risk of allergic diseases is consistently high both before and after INS onset, common environmental triggers, genetic factors, and aberrant immunological responses might contribute to the development of both disorders. Therefore, we conducted a nationwide population-based cohort study to investigate the development of common allergic diseases,^4^^,^^23^ including allergic conjunctivitis (AC), AR, asthma, and AD, in children who were diagnosed with INS.

## METHODS

### Data sources

The National Health Insurance Research Database (NHIRD), maintained by the National Health Research Institutes, is population-based and derived from the claims data of the National Health Insurance program, a mandatory-enrollment, single-payment system created in 1995, now covering over 99% of Taiwan’s population (http://www.nhi.gov.tw/english/index.aspx).^24^ This study used the children file derived from the NHIRD, containing information on half of all children randomly selected from the entire insured population in Taiwan. The dataset contained all medical claims and information on the insured patients and provided a sufficient sample size to pursue the objectives addressed in this study. Because of personal electronic data-privacy regulations, the identification of the insured patients was encrypted before being sent to researchers. Although the identification was scrambled, the study was also approved by the Institutional Review Board of China Medical University Hospital (CMU-REC-101-012). The International Classification of Disease, Ninth Revision (ICD-9-CM) was used to define diagnostic disease codes.

### Study subjects

**Figure 1.**
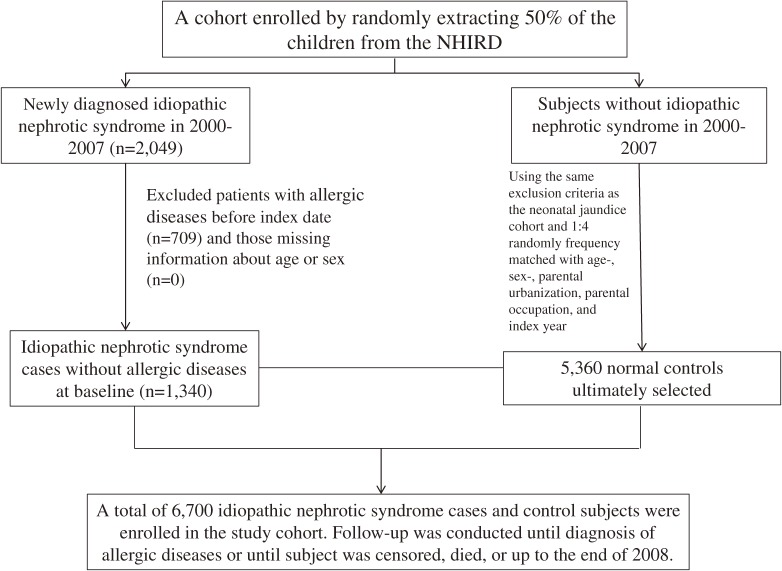
Flow diagram of participant selection.

The diagnosis of INS has been discussed in our previous studies (ICD-9-CM codes 581.3 and 581.9).^23^^,^^24^ Patients being diagnosed with secondary nephrotic syndrome with ICD-9-CM code 581.8 were excluded. One month after the first date of INS diagnosis was the baseline index date because some allergic disorders might already exist when INS is identified. For each child with INS, we randomly selected four frequency-matched non-INS children (frequency matched by sex, age, urbanization of residential area, parental occupation, and baseline year). To improve diagnostic accuracy and account for the chronic relapsing nature of allergic diseases, only those with at least two consecutive corresponding diagnoses were designated as having a given allergic disease. We evaluated the atopic diseases of AC (ICD-9 code 372.05, 372.10, and 372.14), AR (ICD-9 code: 477), asthma (ICD-9 code 493), and AD (ICD-9 code 691.8). Patients with a history of AC, AR, asthma, and AD before the baseline, or those with incomplete age or sex information, were excluded. INS and non-INS cohorts were followed up until the atopic events appeared or until cases were censored because of follow-up failure, death, or the end of 2008 being reached. A flow diagram of the selection process is illustrated in Figure 1.

### Statistical analysis

We first examined the demographic data between INS and non-INS cohorts using a Chi-square test. Continuous data were presented as mean ± standard deviation (SD) and examined using a *t*-test. To assess the difference in the atopic-event-free rates between the two cohorts, the Kaplan-Meier method and log-rank test were applied. We calculated the incidence rates (IRs) of atopic events in both cohorts. The multivariate Cox proportional hazards model was used to calculate the hazard ratio (HR) and 95% confidence interval (CI) of developing an atopic event in association with NS. The multivariate Cox model was simultaneously adjusted for age, sex, and frequency of annual medical visits. This study used SAS software (version 9.2 for windows; SAS Institute Inc., Cary, NC, USA) to perform all data analyses. The graph of the Kaplan-Meier plot was utilized to estimate the cumulative incidence using R software (version 2.14.1; R Development Core Team, Vienna, Austria). All statistical tests were set at a two-tailed significance level of 0.05.

## RESULTS

This study included a cohort of 1340 children with INS (INS cohort) and a cohort of 5360 children without INS (non-INS cohort) (Table 1), and boys predominated in both cohorts. The INS cohort was slightly older than the non-INS cohort. More than half of the children lived in more urbanized areas, and 52.8% of the parents had white-collar jobs. The children with INS visited doctors more frequently in the preceding years before enrollment than those without INS (average annual visits = 15.7 [SD, 24.2] vs 4.37 [SD, 9.45]).

**Table 1.** Demographic characteristics of idiopathic nephrotic syndrome (INS) cohort and non-INS cohort

	Non-INS *N* = 5360	INS *N* = 1340	*P* value
	*n* (%)	*n* (%)	
Sex			0.97
Female	1988 (37.1)	497 (37.1)	
Male	3372 (62.9)	843 (62.9)	
Age, years			0.96
≤5	1708 (31.9)	427 (31.9)	
6–9	1016 (19.0)	254 (19.0)	
10–18	2636 (49.2)	659 (49.2)	
Mean (SD)	9.30 (5.60)	9.43 (5.54)	0.44^a^
Frequency of medical visits, mean (SD)^b^	4.37 (9.45)	15.7 (24.2)	<0.001^a^
Urbanization			0.95
1 (highest)	1180 (22.2)	295 (22.2)	
2	1648 (31.0)	412 (31.0)	
3	1060 (20.0)	265 (20.0)	
4 (lowest)	1424 (26.8)	356 (26.8)	
Occupation			0.97
White collar	2828 (52.8)	707 (52.8)	
Blue collar	1768 (33.0)	442 (33.0)	
Others	764 (14.3)	191 (14.3)	

SD, standard deviation.

Chi-square test, ^a^*t*-test.

^b^Average frequency of medical visits/per year in the study period.

Table 2 shows that the incidences of all five types of allergic disease were greater for the INS cohort than the non-INS cohort in all age groups. The largest difference in disease type-specific incidence was for AR (31.2 vs 9.61 per 1000 person-years for the INS cohort and non-INS cohort, respectively) and the smallest difference was for AD (4.59 vs 1.01 per 1000 person-years for the INS cohort and non-INS cohort, respectively). The incidence of each atopic type decreased as age increased for children in both cohorts. The INS children ≤5 years of age had the highest incidence of AR (54.4 per 1000 person-years). Compared with children aged 10–18 years, those aged ≤5 years had higher adjusted HRs of allergic disorder that ranged from 1.33 (95% CI, 1.01–1.74) for AR to 4.29 (95% CI, 2.29–8.05) for AD. Comparisons of boys and girls showed that the incidences of AC, AR, and asthma were higher in boys in both cohorts, whereas that of AD was higher in girls (Table 3). Boys also had higher adjusted HRs of all allergic disorders except for AD than girls.

**Table 2.** Age-specific incidence rates and hazard ratios of allergic diseases in idiopathic nephrotic syndrome (INS) cohort to non-INS cohort

Allergic disease/ age, year	Non-INS			INS			aHR^a^ (95% CI)	aHR^b^ (95% CI)
	
	Event	PY	IR	Event	PY	IR		
Allergic conjunctivitis								
≤5	107	9415	11.40	51	2213	23.10	2.02 (1.44, 2.81)***	1.69 (1.19, 2.41)**
6–9	45	5824	7.73	25	1388	18.00	2.30 (1.41, 3.75)***	0.69 (0.35, 1.35)
10–18	22	14 636	1.50	8	3593	2.23	1.47 (0.65, 3.30)	0.59 (0.21, 1.70)
** All**	**174**	**29 875**	**5.82**	**84**	**7195**	**11.70**	1.99 (1.54, 2.58)***	1.73 (1.31, 2.28)***
Allergic rhinitis								
≤5	167	9164	18.20	104	1913	54.40	2.88 (2.25, 2.68)***	1.33 (1.01, 1.74)*
6–9	64	5766	11.10	57	1200	47.50	4.11 (2.87, 5.87)***	1.23 (0.77, 1.97)
10–18	52	14 508	3.58	42	3399	12.40	3.37 (2.25, 5.07)***	2.04 (1.30, 3.22)**
** All**	**283**	**29 438**	**9.61**	**203**	**6512**	**31.20**	3.15 (2.63, 3.78)***	1.71 (1.39, 2.09)***
Asthma								
≤5	66	9584	6.89	43	2213	19.40	2.78 (1.89, 4.08)***	1.72 (1.15, 2.57)**
6–9	10	6016	1.66	14	1418	9.87	5.78 (2.57, 13.0)***	3.61 (1.47, 8.82)**
10–18	3	14 755	0.20	1	3630	0.28	1.34 (0.14, 12.9)	0.78 (0.06, 10.9)
** All**	**79**	**30 355**	**2.60**	**58**	**7262**	**7.99**	3.03 (2.16, 4.26)***	1.96 (1.37, 2.80)***
Atopic dermatitis								
≤5	25	9732	2.57	21	2338	8.98	3.44 (1.93, 6.15)***	4.29 (2.29, 8.05)***
6–9	3	6052	0.50	7	1476	4.74	9.51 (2.46, 36.8)**	5.30 (1.20, 23.3)*
10–18	3	14 763	0.20	6	3595	1.67	8.12 (2.03, 32.5)**	1.42 (0.20, 10.2)
** All**	**31**	**30 549**	**1.01**	**34**	**7408**	**4.59**	4.47 (2.75, 7.27)***	4.13 (2.50, 6.83)***

aHR, adjusted hazard ratio; CI, confidence interval; PY, person-years; IR, incidence rate per 1000 person-years.

^a^Adjusted for age and sex.

^b^Adjusted for age, sex, and frequency of medical visits.

*P < 0.05, **P < 0.01, ***P < 0.001.

**Table 3.** Gender-specific incidence rates and hazard ratios of allergic diseases in idiopathic nephrotic syndrome (INS) cohort to non-INS cohort

Allergic disease/ Gender	Non-INS			INS			aHR^a^ (95% CI)	aHR^b^ (95% CI)
	
	Event	PY	IR	Event	PY	IR		
Allergic conjunctivitis								
Girls	49	10 841	4.52	29	2587	11.20	2.45 (1.54, 3.89)*	1.15 (0.65, 2.03)
Boys	125	19 034	6.57	55	4608	11.90	1.94 (1.41, 2.67)*	1.60 (1.14, 2.24)*
Allergic rhinitis								
Girls	82	10 716	7.65	67	2383	28.10	3.82 (2.76, 5.28)*	1.70 (1.18, 2.46)*
Boys	201	18 722	10.70	136	4129	32.90	3.20 (2.57, 3.98)*	1.63 (1.28, 2.08)**
Asthma								
Girls	15	11 018	1.36	17	2631	6.46	5.14 (2.56, 10.3)*	1.66 (0.73, 3.77)
Boys	64	19 337	3.31	41	4631	8.85	2.95 (1.99, 4.38)*	1.81 (1.20, 2.74)*
Atopic dermatitis								
Girls	8	11 047	0.72	15	2648	5.66	8.19 (3.47, 19.3)*	4.83 (1.86, 12.5)*
Boys	23	19 502	1.18	19	4760	3.99	3.73 (2.02, 6.86)*	3.16 (1.67, 5.96)**

aHR, adjusted hazard ratio; CI, confidence interval; PY, person-years; IR, incidence rate per 1000 person-years.

^a^Adjusted for age and sex.

^b^Adjusted for age, sex, and frequency of medical visits.

*P < 0.01, **P < 0.001.

**Figure 2.**
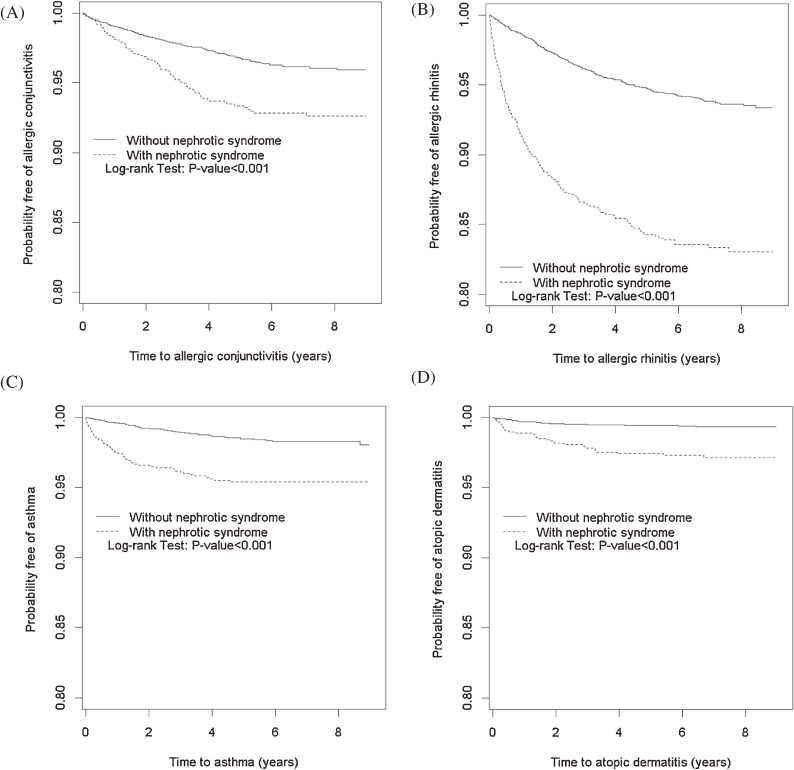
Probability of being free of allergic conjunctivitis (A), allergic rhinitis (B), asthma (C), and atopic dermatitis (D) for patients with (dashed line) and without (solid line) idiopathic nephrotic syndrome.

The Kaplan-Meier plots in Figure 2 show the 9-year probabilities of developing AC, AR, AD, and asthma. The cumulative incidence of each allergic disorder was greater in the INS cohort than in the non-INS cohort (log-rank test, *P* < 0.001). Table 4 shows that most of the allergic disorders appeared within 2–6 months after the INS diagnosis. Among disorders, the incidence of AR was the highest (122.2 per 1000 person-years), which was approximately eight times the incidence in the non-INS cohort (15.0 per 1000 person-years), with an adjusted HR of 4.06 (95% CI, 2.70–6.10). The adjusted HR decreased with time to 0.56 (95% CI, 0.21–1.46) by the end of follow-up. In contrast, the AC incidence was higher after 7–12 months in the INS cohort.

**Table 4.** Follow-up time-specific incidence rates and hazard ratios of allergic diseases in idiopathic nephrotic syndrome (INS) cohort to non-INS cohort

Follow-up time	Non-INS			INS			aHR^a^ (95% CI)	aHR^b^ (95% CI)
	
	Event	PY	IR	Event	PY	IR		
Allergic conjunctivitis								
2–6 months	31	2668	11.60	11	666	16.50	1.45 (0.73, 2.89)	0.84 (0.29, 2.41)
7–12 months	19	2652	7.16	14	654	21.40	3.10 (1.55, 6.18)**	2.63 (1.29, 5.38)**
1–2 years	36	5087	7.08	16	1243	12.90	1.88 (1.04, 3.39)*	1.56 (0.84, 2.90)
3–5 years	66	12 330	5.35	38	2939	12.90	2.59 (1.74, 3.87)***	2.18 (1.43, 3.31)***
5+ years	22	7135	3.08	5	1692	2.95	1.08 (0.41, 2.86)	0.84 (0.29, 2.41)
Allergic rhinitis								
2–6 months	40	2668	15.00	79	646	122.20	8.34 (5.70, 12.2)***	4.06 (2.70, 6.10)***
7–12 months	31	2644	11.70	36	615	58.60	5.16 (3.19, 8.34)***	2.79 (1.64, 4.73)***
1–2 years	71	5048	14.10	39	1142	34.20	2.61 (1.76, 3.85)***	1.27 (0.82, 1.99)
3–5 years	110	12 106	9.09	42	2644	15.90	1.88 (1.32, 2.69)***	1.00 (0.67, 1.50)
5+ years	31	6973	4.45	7	1465	4.78	1.23 (0.54, 2.80)	0.56 (0.21, 1.48)
Asthma								
2–6 months	12	2674	4.49	22	661	33.30	7.67 (3.80, 15.5)***	4.42 (2.12, 9.22)***
7–12 months	10	2664	3.75	11	649	17.00	4.92 (2.09, 11.6)***	3.04 (1.26, 7.32)*
1–2 years	19	5124	3.71	12	1236	9.71	3.02 (1.46, 6.24)**	1.96 (0.93, 4.12)
3–5 years	32	12 517	2.56	13	2976	4.37	1.96 (1.03, 3.75)*	1.06 (0.52, 2.16)
5+ years	6	7376	0.81	0	1740	0.00	—	—
Atopic dermatitis								
2–6 months	8	2675	2.99	13	665	19.50	7.37 (3.04, 17.9)***	6.11 (2.46, 15.2)***
7–12 months	8	2666	3.00	2	666	3.05	1.21 (0.24, 5.30)	0.86 (0.16, 4.59)
1–2 years	7	5136	1.36	9	1259	7.15	5.46 (2.03, 14.7)***	4.95 (1.80, 13.6)**
3–5 years	6	12 610	0.48	8	3039	2.63	5.85 (2.03, 16.9)**	5.02 (1.69, 14.9)***
5+ years	2	7461	0.27	2	1789	1.12	4.32 (0.61, 30.7)	3.68 (0.48, 28.1)

aHR, adjusted hazard ratio; CI, confidence interval; PY, person-years; IR, incidence rate per 1000 person-years.

^a^Adjusted for age and sex.

^b^Adjusted for age, sex, and frequency of medical visits.

*P < 0.05, **P < 0.01, ***P < 0.001.

## DISCUSSION

To our knowledge, this is the first population-based cohort study to precisely quantify the atopic risk of common allergic diseases in children with INS compared with non-INS controls. Most previous studies were anecdotal reports or cross-sectional case-control studies with limited numbers of patients that described the clinical features and immunological findings of certain allergic diseases in INS but failed to confirm whether the atopic disorders occur after INS onset.^1^^–^^9^ The current population-based cohort study may have minimized the selection bias compared to previous case-control studies, while the diagnosis of allergic diseases by physicians instead of via questionnaire minimized the selection bias and recall bias in this retrospective study. Further, the INS and non-INS cohorts were established by matching sociodemographic factors, which may also better control the confounding effect of these factors when measuring risk differences. Our study findings support the relationships between pre-existing INS and the subsequent risk of allergic disorders, including AC, AR, AD, and asthma. These increased risks were consistent for all five disorders across the strata of sex and age.

In this study, the sex-specific relative hazards were higher for girls in all types of disorders, indicating that the impact of INS is greater for girls than for boys. However, the HRs were not different because the incidences were all higher in boys in both cohorts. The incidence of each type of allergic disease is generally inversely associated with age, indicating that the risk declines as children grow. The discrepancy between sexes and age groups was not well documented in previous studies.^1^^–^^9^ The effect was stronger for AR than for other allergic disorders. Most of the allergic disorders appeared within 6 months after the INS developed, and the incidences declined with increasing duration of follow-up. The pathophysiological complexity between INS and all types of allergic consequences warrants further exploration.

The global increase in the prevalence of allergic diseases in recent years has become a serious public health issue.^25^ Allergic diseases develop in part from innate and adaptive immune functional dysregulation, particularly an imbalance in the Th2-driven adaptive immune response.^26^ This dysregulation results from the complex interactions between genes and environment and might be initiated early in life.^20^ Findings from several longitudinal studies suggest that approximately half of patients with AD will develop asthma, particularly those with severe AD, while two-thirds will develop AR.^15^ Cutaneous sensitization and subsequent migration of sensitized T cells to the nose and airways are thought to cause upper and lower airway allergic disease.^15^ A previous study on children with AD showed that this population was at higher risk of INS than those without AD. In current study, children with INS had consistently elevated risks of developing all five allergic diseases.^16^ Taken together, these previous findings imply that INS and allergic diseases may share common early-life determinants and a common aberrant immunological response.

INS is commonly thought to represent a spectrum of podocytopathies that result in increased glomerular permeability and massive proteinuria.^27^ An elevated IgE level has been widely reported in children with INS.^6^^–^^14^ A recent cohort study also revealed increased incidence and risk of INS in children with AD.^22^ Allergic disease is a Th2-driven and IgE-mediated chronic inflammation of the target organs. Interleukins 4, 5, and 13 are expressed during Th2 cell activation.^18^^,^^19^ Interleukins 4 and 13 are important in B-cell IgE isotype switching and IgE production; interleukin 13 is also involved in the modulation of eosinophilic inflammation and the recruitment of monocytes and T-cells.^18^^,^^19^ Serum interleukin 5 and 13 levels were higher in patients with steroid-sensitive NS before treatment than after,^20^ and the number of CD3+ interleukin 13-producing cells was increased in nephrotic children experiencing relapse.^26^

Shimada et al recently proposed the “two-hit podocyte immune disorder” hypothesis.^27^^–^^31^ First, CD80 expression is induced in podocytes by circulating cytokines (such as interleukin 13), microbial products, or allergens.^27^^–^^29^ Then, due to regulatory T lymphocyte (T_reg_) dysfunction and/or impaired CD80 autoregulation, sustained podocyte injury results in INS.^29^^–^^31^ T_reg_ cells are essential for maintaining homeostasis of the immune system. Further, T_reg_ cell-mediated suppression serves as a vital mechanism of negative regulation of immune-mediated inflammation and is featured in allergic disorders.^32^^,^^33^ Hence, T_reg_ dysfunction may play an important role in the development of both allergic disorders and INS after environmental exposure to microbial products or allergens. Clinical observations have shown that viral upper respiratory tract infections often precipitate INS relapse and respiratory allergic symptoms. This may also explain why the increased incidence of each type of allergic disease is especially significant within the first year of the diagnosis of INS in the current study. These findings also support a common aberrant immunological response after environmental exposure between allergic disease and INS.

Our study has some limitations. Detailed clinical information, such as laboratory data and environmental exposures, were unavailable in the claims data, so their roles were not examined. Children with INS are likely to visit their physicians more often and have an allergic disease diagnosed earlier than children without INS. Hence, in this study, we also adjusted the frequency of medical visits for the risk of allergic diseases and found consistently increased risks of developing four allergic diseases. Corticosteroids are the first-line therapy for INS,^34^ and their systemic anti-inflammatory properties potentially decrease the occurrence of atopic disorders; however, our findings conversely found an increased incidence of such disorders among patients receiving corticosteroids, which further supports the strong association between INS and allergy.

In conclusion, this study provides better insight into the relationships between childhood INS and subsequent allergic disorders. These may appear soon after INS is diagnosed, especially in young children. Much more effort should be made to clarify the correlations among these conditions.
